# Analysis of a novel hydrophobic acrylic enhanced monofocal intraocular lens compared to its standard monofocal type on the optical bench

**DOI:** 10.1186/s12886-022-02584-8

**Published:** 2022-09-03

**Authors:** A. F. Borkenstein, E. M. Borkenstein, R. Schmid

**Affiliations:** 1 Borkenstein and Borkenstein Private Practice, Privatklinik Kreuzschwestern Graz, Kreuzgasse 35, 8010 Graz, Austria; 2 Accuratis, Practice for Refractive Eye Surgery, Hirschstrasse 1, 89073 Ulm, Germany

**Keywords:** Enhanced monofocal intraocular lens, Optical bench study, Laboratory study, Quality of intraocular lens

## Abstract

**Introduction:**

The aim of this laboratory study is to objectively analyze the new hydrophobic, acrylic, enhanced monofocal intraocular lens Acunex Quantum (AN6Q) and compare it with the monofocal platform Acunex AN6.

**Methods:**

Two IOL models were analyzed (Acunex Quantum AN6Q and Acunex AN6, Teleon Surgical, Spankeren, Netherlands), each having the same refractive power of + 22.0 D, on the optical bench with the OptiSpheric IOL PRO 2. The measurements followed the guidelines of the International Standard Organization with following parameters: ISO 2 cornea (+ 0,28 µ), ISO 11979/2, lens placement in situ in NaCl with 35° temperature, 546 nm and selection of different aperture sizes (3.0 mm vs 4.5 mm). The aberrations of each IOL were evaluated by the WaveMaster IOL 2, a high-resolution Shack-Hartmann sensor in reverse projection setup. An in-situ model eye was used according to ISO 11979 in NaCl (*n* = 1.337) with 546 nm, mask width 4.51. Zernike polynomials up to 10th order were determined by means of the measured wavefront that describe the optical properties of the IOL.

**Results:**

Through frequency modulation transfer function (mean) at 50 lp/mm (AN6Q/AN6 centered) was 0.687/0.731 (3.0 mm aperture) and 0.400/0.509 (4.5 mm aperture). The SR (mean) was 0.592/0.809 (3.0 mm) and 0.332/0.372 (4.5 mm). The MTF (mean) at 50 lp/mm (AN6Q/AN6 decentered by 1 mm) was 0.413/0.478 (3.0 mm) and 0.257/0.229 (4.5 mm). The SR (mean) was 0.393/0.404 (3.0 mm) and 0.183/0.212 (4.5 mm). The MTF (mean) at 50 lp/mm (AN6Q/AN6 tilted by 5°) was 0.508/0.710 (3.0 mm) and 0.337/0.513 (4.5 mm). The SR (mean) was 0.508/0.760 (3.0 mm) and 0.235/0.2372 (4.5 mm). AN6Q showed MTF peak of 0.55 with an enlarged depth of power of about 2.5 D and two cusps in the MTF curve. The spherical aberration Z 4–0 was about -0.21 µm and the secondary spherical aberration Z 6–0 was about 0.16 µm. No other relevant aberration showed up.

**Conclusion:**

The new, enhanced monofocal AN6Q provides an extended range of focus with only slight decrease in contrast quality. Both types of the hydrophobic, acrylic Acunex IOL platform have its particular advantages in clinical settings and therefore its importance, respectively.

## Introduction

Recently, studies found that patients after cataract surgery with enhanced monofocal intraocular lenses (IOL) reported a better performance in activities that require intermediate vision [1–3]. These enhanced monofocal IOLs represent a very new type of IOLs in the market and can provide intermediate vision, and, at the same time similar far distance performance and photic phenomena compared with standard monofocal IOLs. By that, functional performance in daily life will be improved [4, 5].

These improvements and developments in monofocal IOL technology also offer new options to improve visual function in cases of multifocal IOL explantation due to adverse events and side effects of dysphotopsia like halo/glare [6, 7].

A variety of new and innovative lens designs have recently entered the market and the few studies made up to date are showing positive and promising clinical data.

However, in addition to clinical trials and long-term results, it seems also mandatory to evaluate new IOLs and their optical principles on the optical bench in a setting independent from IOL companies. This will help the clinician to better understand the optical properties of an IOL and, by that, to better decide which IOL to implant in a specific cataract case.

The aim of this study is to objectively analyze the new IOL Acunex Quantum (AN6Q) and compare it with the monofocal platform Acunex AN6.

**Fig. 1:**
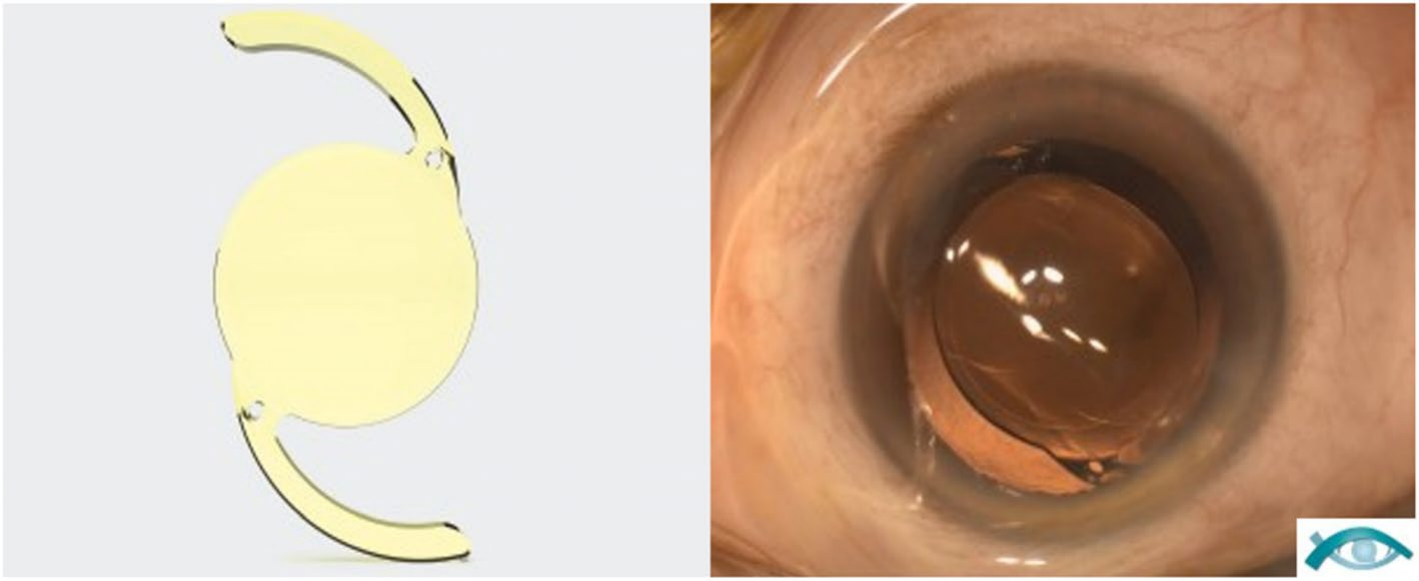
Acunex by Teleon Surgical (Spankeren, Netherlands) is a hydrophobic, aberration correcting, foldable, one-piece intraocular lens. The overall diameter is 12.5 mm and the optic diameter is 6.0 mm (left). The material has a high refractive index of 1.54. Slit lamp image showing the enhanced monofocal AN6Q well positioned in the capsular bag (right)

The Acunex Quantum (AN6Q) by Teleon Surgical (Spankeren Netherlands) is a one-piece, foldable, hydrophobic acrylic IOL with a spherical aberration correction of -0.13 µm on the posterior surface (Fig. 1). The lens has a step vaulted C-loop design, an optical diameter of 6.0 mm and an overall diameter of 12.5 mm. The UV absorbing material with a water content of 4% has a high refractive index of 1.54 and a blue light filter is incorporated. A subtle modification of the very central part of the optics of the AN6Q (named “Q-Zone technology” by the manufacturer) is applied to increase depth of focus. Otherwise, material and basic geometry are identical to the monofocal counterpart, the Acunex IOL (AN6).

## Methods

Two IOL models were analyzed (Acunex AN6 and Acunex Quantum AN6Q, Teleon Surgical, Spankeren, Netherlands), each having the same refractive power of + 22.0 D, on the optical bench with the OptiSpheric IOL PRO 2 and the Wavemaster IOL 2 (Trioptics company, Wedel, Germany).

All measurements were performed independently by Trioptics company on its own optical bench by a mandated, specialized optician who did not know the aim of the study. By that, an independent analysis was guaranteed. Results were confirmed with official certificates.

The measurements followed the guidelines of the International Standard Organization. The OptiSpheric IOL PRO 2 (Trioptics, Wedel, Germany) device was used to assess the optical quality of the two IOL models (AN6 and AN6Q). The following parameters were used: ISO 2 cornea (+ 0,28 µ), ISO 11979/2, lens placement in situ in NaCl (*n* = 1.337 solution index) with 35° temperature, 546 nm and selection of different aperture sizes (3.0 mm vs 4.5 mm). All measurements were repeated 3 times and performed on 2 different lenses of the same type. For each measurement, 5 tangential and 5 sagittal measurements were obtained and averaged. For rotational symmetrical IOLs, tangential and sagittal values generally are rather identical. The following settings were used: centered IOL, decentered IOL (1.0 mm) and tilted IOL (5°).

To simulate photopic and mesopic pupil conditions, measurements were performed with 3.0 mm and 4.5 mm aperture sizes. (Note: ISO standard 11,979–2 specifies MTF measurement at 1 mm decenter and 5 degrees tilt (annex C.7).

The following parameters were measured with the OptiSpheric IOL PRO 2 as the main criteria to describe the quality of the intraocular lens: The modulation transfer function (MTF) describes the contrast sensitivity of a lens system and is the imaging power of a lens at different spatial frequencies in the tangential and sagittal directions. Through frequency MTF were obtained for each IOL and each alignment. The Strehl number reflects the overall optical performance of an IOL as it takes into account the small oscillations that occur on the MTF curve. The Strehl Ratio (SR) is a measure of the imaging quality of an optical system over its entire spatial frequency range compared to the corresponding ideal (diffraction-limited) system.

Through focus MTF at 50 linepairs/mm were obtained additionally. To compare the depth-of-focus performance of both IOLs, an autofocus scan of the IOL was done. The through focus performance was defined as the distance between the foci of diopter powers showing a MTF better than 0.1.

The aberrations of each IOL were evaluated by the WaveMaster IOL 2 (Trioptics, Wedel, Germany), a high-resolution Shack-Hartmann sensor in reverse projection setup. An in-situ model eye was used according to ISO 11979 in NaCl (*n* = 1.337 solution index) with 546 nm, mask width 4.51, corresponding to standard settings and to a mesopic pupil. Zernike polynomials up to 10^th^ order were determined by means of the measured wavefront that describe the optical properties of the IOL. It should be noted that spherical aberrations will be caused mainly by the lens’ optical design, while asymmetric aberrations such as Coma or Trifoil may partly result from lens errors. Zernike coefficients greater than 0.1 µm were defined as optical relevant.

## Results

### Through frequency MTF

**Fig. 2:**
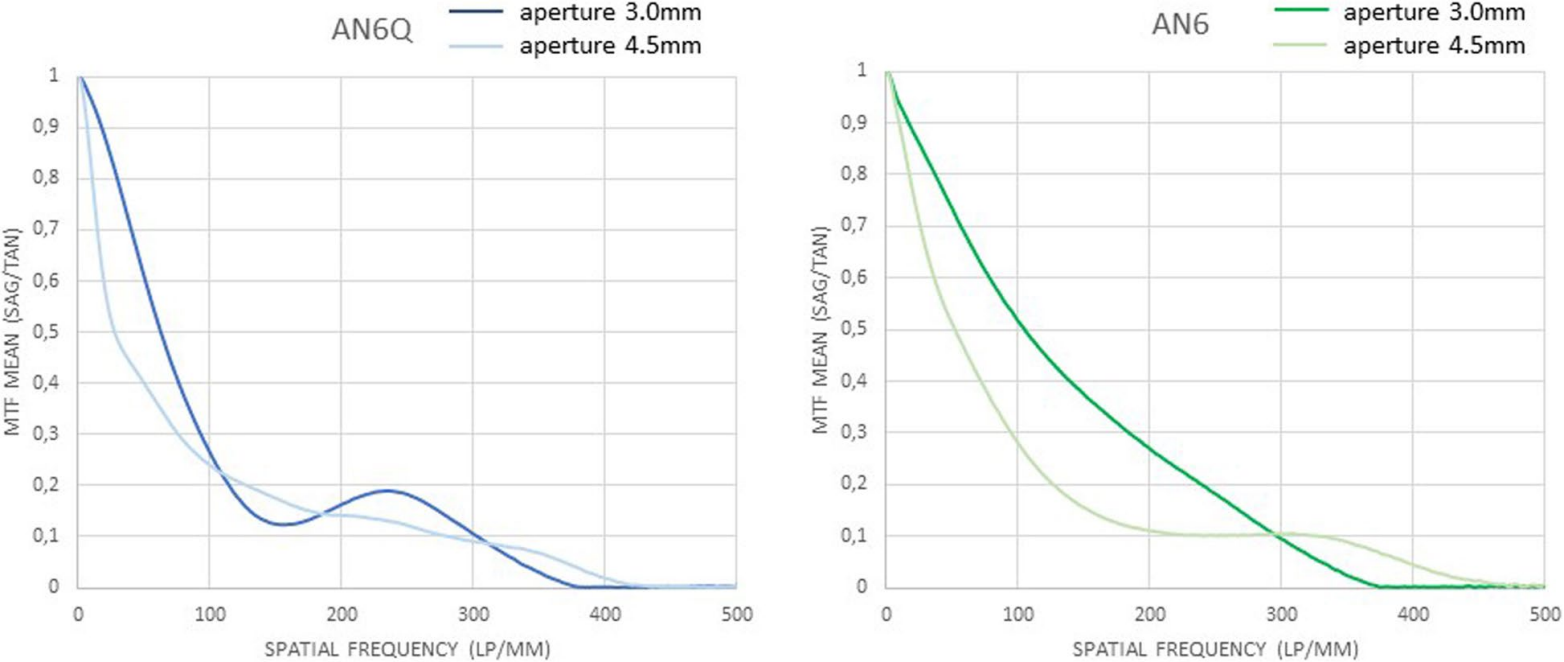
Through Frequency modulation transfer function (MTF mean) curves of centered AN6Q lens (left) and AN6 lens (right) with 3.0 mm aperture and 4.5 mm aperture

**Fig. 3:**
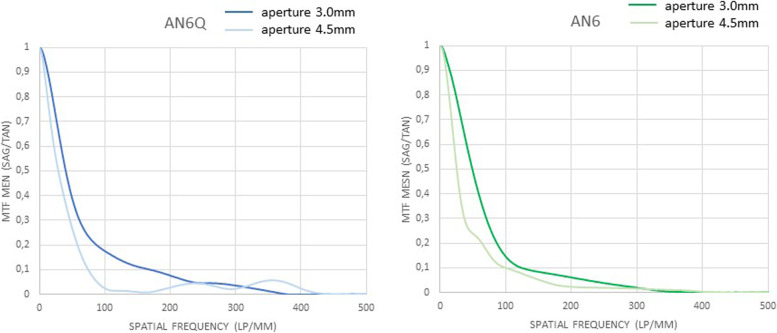
Through frequency modulation transfer function (MTF mean) curves of decentered AN6Q lens (left) and AN6 lens (right) with 3.0 mm aperture and 4.5 mm aperture

**Fig. 4:**
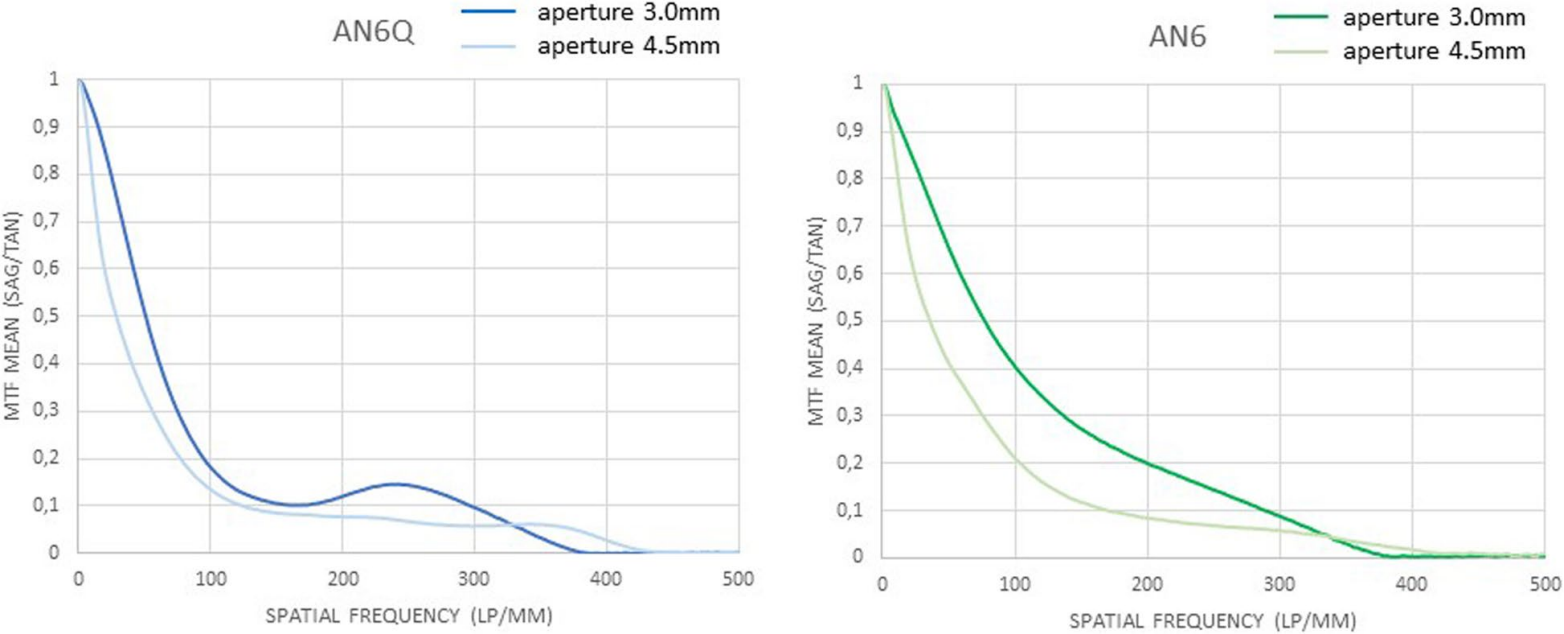
Through frequency modulation transfer function (MTF mean) curves of tilted AN6Q lens (left) and AN6 lens (right) with 3.0 mm aperture and 4.5 mm aperture

The through frequency modulation transfer function of all tested lenses, measured at the best focus through the 3.0 mm aperture (subsequently called “small”) and 4.5 mm aperture (subsequently called “large”), are shown (Figs. 2, 3 and 4). Centered: The through frequency modulation transfer function (MTF-mean) at 50 lp/mm (AN6Q/AN6) with small aperture was 0.687/0.731 and with large aperture 0.400/0.509. The Strehl Ratio (SR-mean) with small aperture was 0.592/0.809 and with large aperture 0.332/0.372. Decentered by 1 mm: The through frequency modulation transfer function (MTF-mean) at 50 lp/mm (AN6Q/AN6) with small aperture was 0.413/0.478 and with large aperture 0.257/0.229. The Strehl Ratio (SR-mean) with small aperture was 0.393/0.404 and with large aperture 0.183/0.212. Tilted by 5°: The through frequency modulation transfer function (MTF-mean) at 50 lp/mm (AN6Q/AN6) with small aperture was 0.508/0.710 and with large aperture 0.337/0.513. The Strehl Ratio (SR-mean) with small aperture was 0.508/0.760 and with large aperture 0.235/0.2372. Strehl findings are in accordance with MTF and all results are summarized in Tables 1 and 2.

**Table 1:**
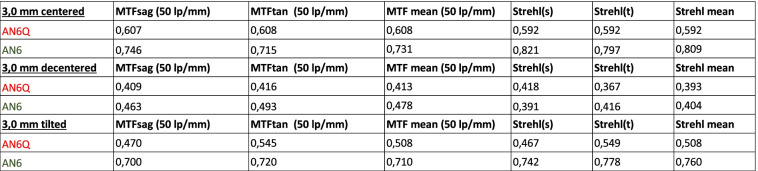
Showing data of MTF and Strehl with aperture of 3.0 mm and centered, decentered (1 mm) and tilted (5°) IOLs

**Table 2:**
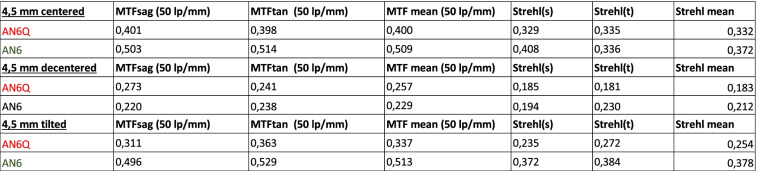
Showing data of MTF and Strehl with aperture of 4.5 mm and centered, decentered (1 mm) and tilted (5°) IOLs

### Through focus MTF

**Fig. 5:**
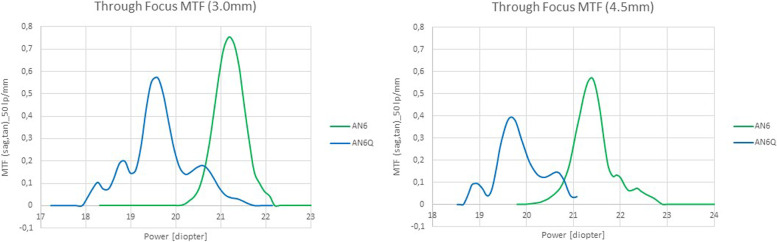
Through Focus modulation transfer function with 3.0 mm aperture (left) and 4.5 mm aperture (right) of the enhanced monofocal Acunex Quantum (AN6Q) and the monofocal Acunex (AN6). Note to Fig. 5: IOLs of the same power were measured, the shift on the x-axis can be explained as follows: It can have an influence on how long the IOL is in the model eye. Although the model eye is heated, it is still possible that one lens is measured immediately after insertion and the second lens in another model eye is in situ a little bit longer before the measurement is automatically completed in the device. Temperature can have an influence on the measured power (x-axis) but not on the MTF curve itself. During measurement the Stage always moves to the optimal focus position. The MTF is then measured at this correct position. Of course, one could modify the x-axis afterwards and superimpose the curves to “beautify” it, but as this phenomenon is well known and does not change the accuracy of the MTF curve/y-axis itself, it was not done

The through focus results are presented in Fig. 5. For the aperture of 3.0 mm, AN6 showed a pronounced peak in MTF of about 0.72 with a depth of power of 1.25 D. AN6Q showed MTF peak of 0.55 with an enlarged depth of power of about 2.5 D and two cusps in the MTF curve.

As expected, for the aperture of 4.5 mm, the MTF values of AN6 and AN6Q were markedly reduced, with a shape of the through focus curves roughly similar to the aperture of 3 mm. AN6 showed a MTF peak of 0.51 and a depth of power of about 1 D. AN6Q showed a MTF peak of 0.39 and some reduced depth of power of about 1.5 D.

### Wavefront

**Fig. 6:**
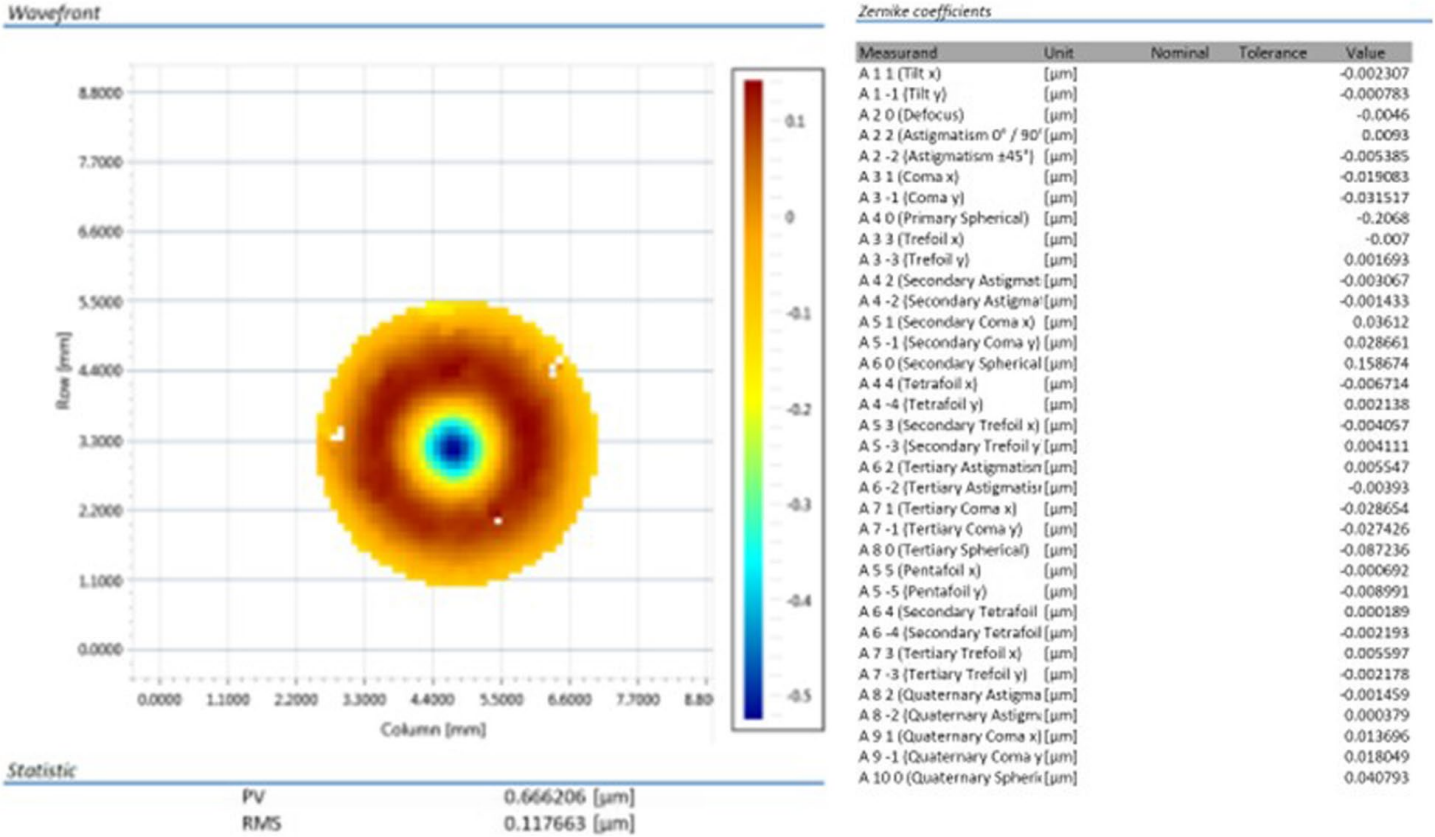
Wavefront mapping of the AN6Q. Overall peak-to-valley (PV) and root mean square (RMS) in µm (left). Measured Zernike coefficients are presented (right). Lower and higher order aberrations up to 10th order obtained. Values more than 0.1 µm are considered as optical relevant

**Fig. 7:**
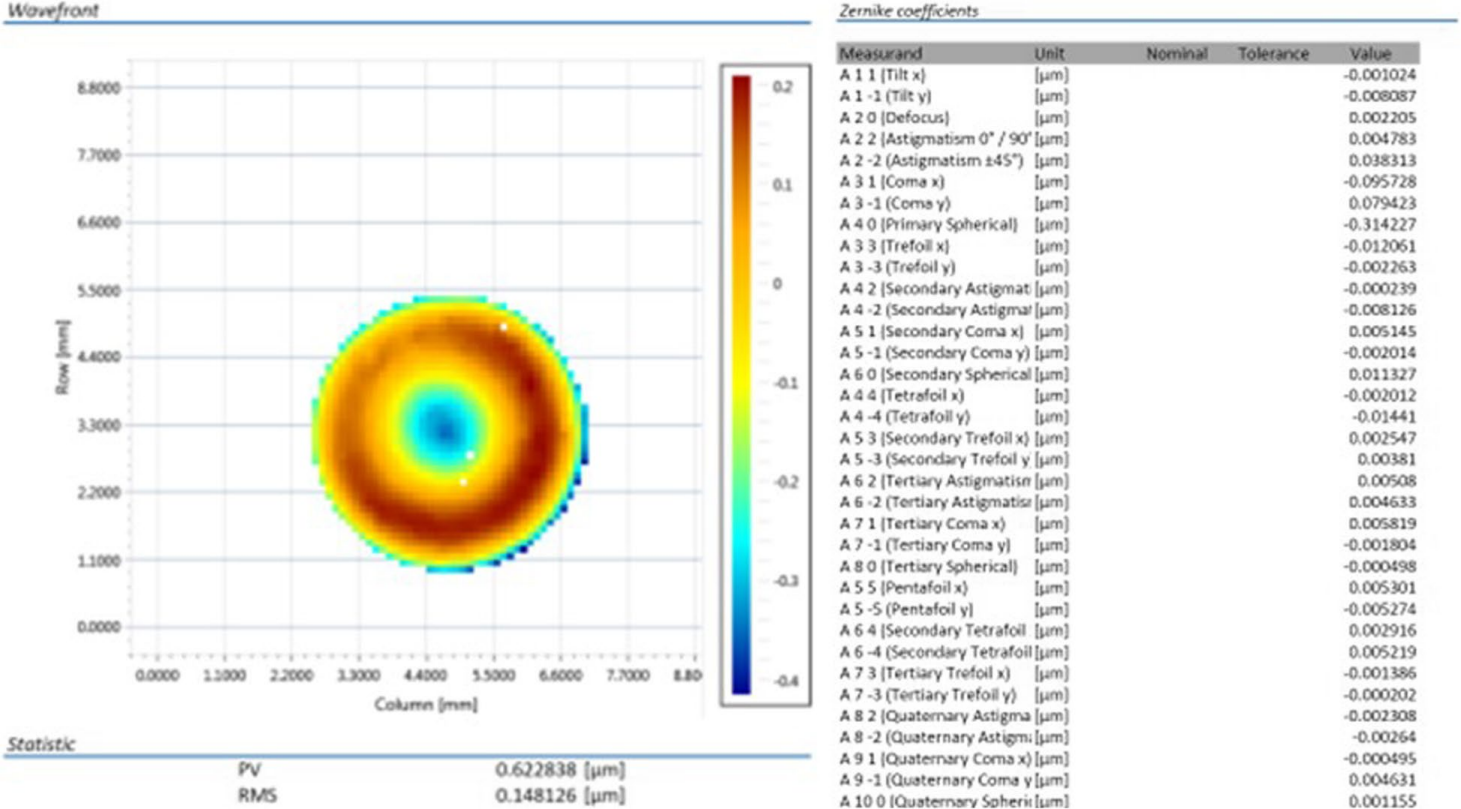
Wavefront mapping of the AN6. Overall peak-to-valley (PV) and root mean square (RMS) in µm (left). Measured Zernike coefficients are presented (right). Lower and higher order aberrations up to 10th order obtained. Values more than 0.1 µm are considered as optical relevant

The wavefront maps and Zernike coefficients of AN6Q and AN6 are presented in Figs. 6 and 7. For Acunex Quantum AN6Q, the spherical aberration Z 4–0 was about -0.21 µm and the secondary spherical aberration Z 6–0 was about 0.16 µm. No other relevant aberration showed up. Peak-to-valley (PV) was 0.67 µm and root mean square (RMS) 0.12 µm.

For Acunex AN6, a spherical aberration Z 4–0 of about -0.31 could be revealed. No other relevant lower order aberrations (LOA) or higher order aberrations (HOA) were detected. Peak-to-valley (PV) is altered by pixel artifacts at the map’s margin.

## Discussion

The small cusp in the through frequency MTF curve of AN6Q represents the modification of the optical surface of this IOL. The monofocal AN6 proved to have a better through frequency MTF compared to AN6Q when centered and tilted. With decentration, both IOLs showed a similar pronounced decrease in MTF.

For the large aperture, corresponding to the mesopic pupil size of an elder person like a cataract patient, the MTF decreased for both IOLs, as expected. The apparently more pronounced decrease of the AN6 with large aperture is due to its very good performance with small aperture when centered or tilted. These results are in accordance with the expectations for our setting. With the positive spherical aberration of + 0.28 of the ISO-2 cornea, the aberration correcting AN6 can provide a very good image contrast.

The large aperture MTF is somewhat degraded because the spherical aberration (SA) of the IOL and model eye do not exactly match. However, real patients have a range of corneal SA, so this is a realistic simulation.

The optics of AN6 obviously are quite robust towards tilt which is advantageous for various anatomies of the patient’s eye, for complicated surgery but also in respect of the natural tilt of the lens and the IOL in the human capsular bag [8, 9].

The transfer of MTF results on the optical bench to clinical contrast visual acuity is always somewhat limited, of course. In clinical studies, there are various corneal asphericities of various patients, different geometries of the eyes, different pupil sizes, neuroadaptation effects and, especially, polychromatic light is present.

The improved depth of focus of AN6Q, claimed by the manufacturer, was confirmed in our study, although somewhat reduced for the big aperture corresponding to larger pupils. This could slightly affect the intermediate vision under mesopic conditions. However, in photopic conditions, a smaller pupil during pupillary near reaction will support the AN6Q IOL to provide a fairly convincing intermediate vision.

We could reveal that the Acunex Quantum AN6Q effectively generates this depth of focus seen in the through focus MTF curves by combining spherical aberration (Z 4–0) and secondary spherical aberration (Z 6–0) of opposite sign. This approach was proposed in literature in the last years and is applied to other novel IOL optics, like LuxSmart IOL (Bausch&Lomb) [10–12].

To know the underlying wavefront patterns of an IOL optics is extremely important for the clinician to understand the function of the particular IOL optics. Generally, no manufacturer will reveal the detailed aberrations of its IOLs. Therefore, our wavefront measurements can furnish important information for cataract surgeons. Wavefront patterns of both IOL are in accordance with the MTF results obtained.

### Limitations of laboratory studies

To transfer the wavefront measurements to clinical settings, it has to be considered that any cornea will induce convergent light on the IOL and thus evoke aberrations of the IOL that are different from the nominal pattern. Furthermore, any laboratory measurement of IOL aberrations is done in air, water or NaCl with a central laser light beam of a defined wavelength. Different wavelengths will produce different wavefront errors. Finally, different diopters of an IOL with different lens thickness and thus material dispersion will produce some different wavefront [9]. We also want to emphasize that only objective laboratory data of the new enhanced monofocal IOL and the monofocal counterpart is presented here trying to give the reader an overview and the chance to compare the two lenses with the same platform. Of course, clinical studies with high case numbers are very important to work out advantages and disadvantages of the different lens models in real life scenario. In addition, one should be careful when making comparisons to other EDoF or enhanced monofocal lenses of competitors with different optical principles.

## Conclusion

The monofocal AN6 can provide a sharp contrast and a distinct image focus, while the enhanced monofocal AN6Q provides an extended range of focus with some minor decrease in contrast quality. Both types of the Acunex IOL platform have its particular advantages in clinical settings and therefore its importance, respectively.

The knowledge about the characteristics of both IOLs on the optical bench can help the cataract surgeon to better decide which type of IOL to implant in a specific patient’s eye up to the individual patient demands and expectations as well as the individual corneal asphericity.

## Data Availability

The authors confirm that the data supporting the findings of this study are available within the article.
